# Investigation of Plasma cell‐free cancer genome chromosomal instability as a tool for targeted minimally invasive biomarkers for primary liver cancer diagnoses

**DOI:** 10.1002/cam4.3142

**Published:** 2020-05-27

**Authors:** Shuang Feng, Zhiwen Ding, Jin Wang, Ziliang Qian, Shanshan Li, Cunzhen Zhang, Haibei Xin, Shupeng Liu, Guanghui Ding, Minggen Hu, Yan Meng, Nan Li

**Affiliations:** ^1^ Department of Radiotherapy Shanghai Eastern Hepatobiliary Surgery Hospital Shanghai China; ^2^ Department of Hepatic Surgery I(Ward I) Shanghai Eastern Hepatobiliary Surgery Hospital Shanghai China; ^3^ Department of Hepatobiliary and Pancreatic Surgical Oncology Chinese PLA General Hospital and Chinese PLA Medical School Beijing China; ^4^ Prophet Genomics Inc San Jose CA USA; ^5^ Changhai Hospital Second Military Medical University Shanghai China

**Keywords:** Cell‐Free DNA, Chromosomal Instability, Primary Liver Cancer

## Abstract

**Purpose:**

To characterize plasma cell‐free cancer genome chromosomal instabilities (CIN) in patients with liver cancer and to evaluate the potential of CIN as minimally invasive biomarkers for primary liver cancer (PLC) diagnoses.

**Experimental Design:**

We collected 196 plasma samples from 172 individuals in two cohorts, a discovery cohort of surgery ineligible PLC patients and a validation cohort of hepatectomy patients with pathological disease confirmations. All samples were subjected to HiSeq X10 sequencing followed by a customized bioinformatics workflow Ultrasensitive Chromosome Aneuploidy Detection (UCAD).

**Results:**

In the discovery cohort, 29 significant copy number changes were identified in plasma from surgery‐ineligible PLC. Twenty‐two (95.7%) surgery‐ineligible liver cancers were identified as harboring copy number changes in at least 1 of 29 segments. Meanwhile 40/41 (97.6%) noncancers harbored no changes. In the validation cohort, 54 (69.4%) surgery‐eligible liver cancers were identified with positive screening, all of which were subsequently confirmed as cancer by pathological examination. Moreover, 26/27 = 96.3% noncancers were identified with negative screening. UCAD‐positive screening was significantly associated with microvascular invasion (OR > 10, 95% CI:[2.53,]), tumor stages B and C (OR = 8.59, 95% CI [1.07, 400]), and tumor size ≥ 3 cm (OR = 5.68, 95% CI [1.43, 28.1]). Furthermore, we collected 29 followed‐up plasma samples from 19 postsurgery patients. Nine (31.0%) postsurgery samples from 6 (31.5%) patients were identified with positive screening. Among them, 3 patients (50.0%) with positive screening were then confirmed as having disease recurrences.

**Conclusions:**

In addition to AFP, plasma cell‐free DNA sequencing is a useful tool for primary liver cancer diagnoses.

## Graphical abstract

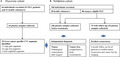

## 1 INTRODUCTION

Primary liver cancer (PLC) is one of the leading causes of cancer‐related death in China.^1^ There are approximately 120 million people with HBV infection history in China^2^ who are at risk for developing PLC. Five‐year overall survival rate remains less than 20% for all liver cancer patients,^1^ although significant improvements have been made in PLC diagnosis and treatments in recent decades. The most important reason for poor survival is relatively advanced stage upon PLC diagnosis. Ultrasound (US) is recommended as the primary screening tool for PLC.^3^ US has a modest sensitivity of approximately 60%, and US screening for PLC is strongly dependent on physician's experience. Computed axial tomography (CT) and magnetic resonance imaging (MRI) are also recommended for high‐risk patients. These screening modalities are associated with increased detection of PLC compared to US. However, they also possess high false‐positives rates. In addition, radiation exposure is also harmful to those who undergoing cancer screening.

Alternatively, blood testing is considered less invasive and less labor‐intensive than the abovementioned screening modalities. AFP measurement is one of the commonly used blood testing approaches for PLC screening.^4^ However, AFP alone is not recommended as an HCC screening test due to its poor sensitivity and specificity. At a serum level of 20 ng/mL, AFP is identified with low sensitivity ranging from 25% to 65% for detecting HCC.^3^ At a serum level of 200ng/ml, only one third of HCC tumors express AFP at levels greater than 200ng/ml.^4^ In addition, patients with chronic liver disease in the absence of malignancy also express AFP.

Chromosome instability (CIN) and gene copy number variations (CNV) were discovered as early events of HCC development. Chromosome 1q and 8q amplifications are frequently reported in HCC tissue profiling studies.^5^ The TCGA HCC dataset shows that greater than 95% of liver cancer samples were identified with at least 5% of the genome amplified or lost.^6^ The data suggest that CIN may serve as a trackable biomarker for HCC management.

Recently, plasma cell‐free DNA (cfDNA) use for prenatal tests has been proven to yield minimal false positives and false negatives.^7^, ^8^ Similar to fetal tissues, tumors also continually shed DNA into the peripheral blood stream. CIN has also been detected in breast cancer,^9^ hepatocellular carcinoma,^5^ lung cancer,^10^ and colorectal cancer.^11^ The characterization of cfDNA in HCC patients has been well reviewed in paper.^12^ Both methylation analyses and genetic analyses of cfDNA show promising results in previous researches. Especially in a recent report,^5^ cfDNA CIN was successfully detected in 87% of hepatocellular carcinoma patients by analyzing chromosome 1 and 8 copy number variations. Through the analysis of cfDNA methylation and genomic studies of cfDNA, it is clear that cfDNA has potential to address clinical questions for the HCC clinical practices. The recent studies have laid the foundation for clinical studies into the use of cfDNA in early detection. However, there are still challenges posed by intratumor genetic heterogeneity, especially when limit biomarkers were investigated.

In addition to chr1 and 8, additional chromosomal segments, such as chr17p,^13^ chr4,^14^ chr11,^6^, ^14^ etc, where TP53(17p13.1) and FGF19(11q13.3) genes are located, were also linked to liver cancer development.^15^, ^16^ Herein, we further investigate these detectable chromosomal changes at the whole genome level in plasma from primary liver cancer patients.

## 2 MATERIALS AND METHODS

### 2.1 Sample collection

One hundred seventy‐two patients were admitted to the Eastern Hepatobiliary Surgery Hospital and Chinese PLA General Hospital. Blood samples were collected for cfDNA extraction. Surgery‐eligible patients were identified by following the “Guidelines for Diagnosis and Treatment of Primary Liver Cancer in China (2017 Edition)”.

### 2.2 Next‐generation sequencing

Total genomic DNA and cfDNA were isolated from tissue samples and plasma using the Amp Genomic DNA Kit (TIANGEN) and QIAseq cfDNA Extraction kit (Qiagen), respectively. Next‐generation sequencing was performed as previously described.^17^, ^18^ DNA was fragmented into an average size of 300bp (cfDNA without fragmentation) and, then, 100 ng of fragmented genomic DNA (cfDNA 10 ng) was used for preparation of sequencing libraries (NEBnext Ultra II). Next, 8 bp barcoded sequencing adaptors were then ligated with DNA fragments and amplified by PCR. Purified sequencing libraries were parallel sequenced by the Illumina HiSeq Xten platform. 4G raw sequencing data per sample were filtered and aligned to the human reference genome.

### 2.3 Ultrasensitive chromosomal aneuploidy detector (UCAD) workflow

Plasma cell‐free DNA was extracted and analyzed using the Illumina X10. At least 10M paired reads were collected for each sample, and the reads were mapped to the human reference genome hg19. Genomic coverage was then counted by using software samtools mpileup.^19^ We then calculated the average coverage for each 200 k bin. Z‐scores for each bin were then normalized to Z‐score by using the following formula:

(1) $${\text{coverage}}_{\text{normalized}}=\frac{{\text{coverege}}_{\text{raw}}-\text{mean}\left({\text{coverage}}_{\text{controls, raw}}\right)}{\text{stdev}\left({\text{coverage}}_{\text{controls, raw}}\right)}.$$

The circular binary segmentation (CBS) algorithm from R package DNACopy^20^ was then used to identify significant genomic breakpoints and copy number changed genomics segments.

R package ‘DNACopy’ was used to analyze copy number changes. A *P* value of <0.05 was considered significant binary segmentation. Absolute segment value was used for further analysis. The sensitivity and specificity of UCAD were estimated by receiver operating characteristic (ROC) curves. For categorical variables, the chi‐square test was used as appropriate. All statistical analyses were performed using SPSS17.0.

Fisher exact tests were used to analyze the associations between clinicopathological UCAD screening positivity and clinicopathological parameters. Data are reported as the means and standard deviations, medians and interquartile ranges, and hazard ratios or odds ratios with 95% confidence intervals, as appropriate. Missing data were removed from the analyses. All analyses were performed with the use of R software, version 3.4.3 (R Foundation for Statistical Computing). Anonymized data and R code used in the statistical analysis will be made available upon request.

The protocol of the study adhered to the tenets of the Declaration of Helsinki and was approved by the local ethics committee.

## 3 RESULTS

### 3.1 Study design

**Figure 1:**
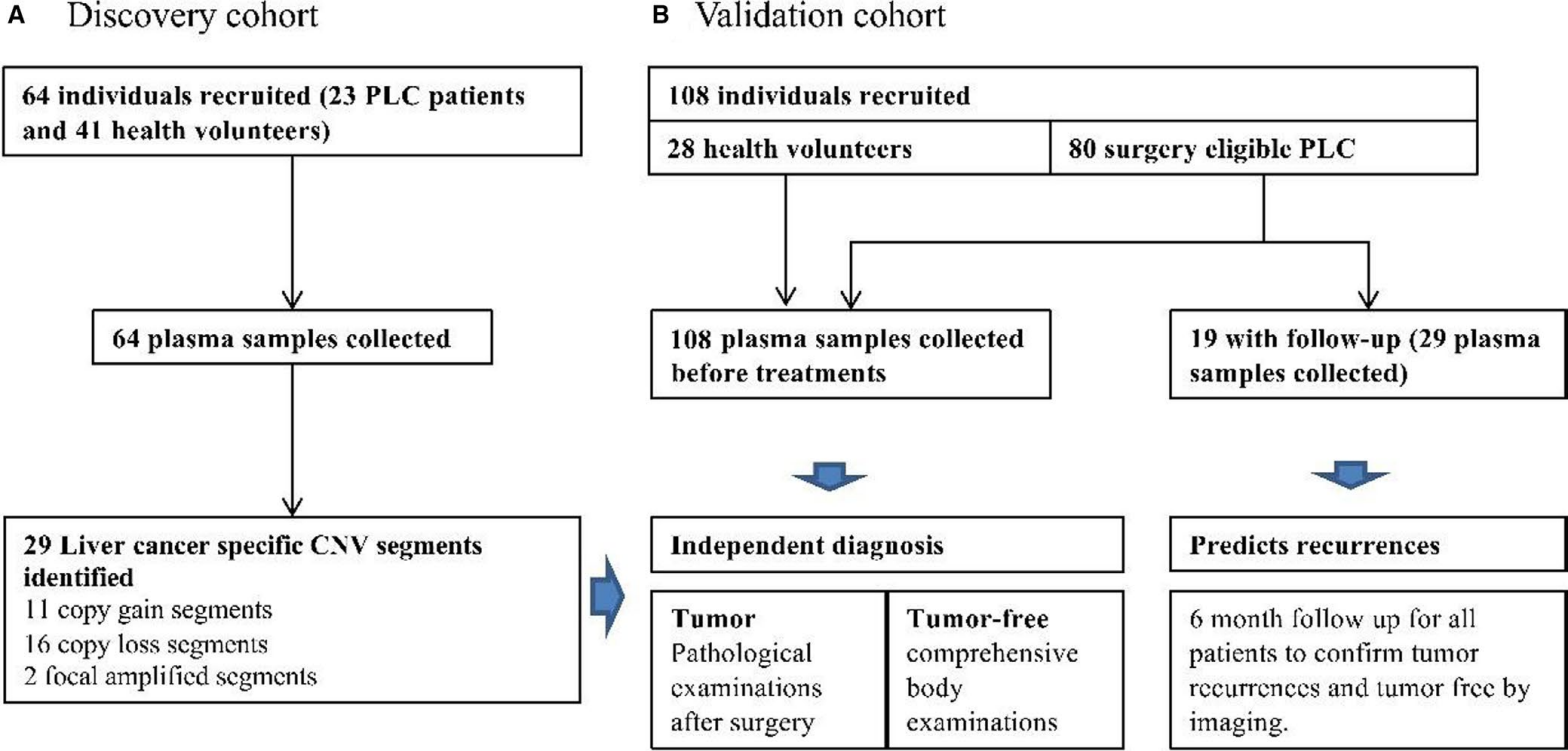
Study design

One hundred ninety‐six fresh plasma samples were collected from 172 individuals (Figure 1) during discovery and validation phases (Figure 1, Table S1). Samples were then processed according to the ultrasensitive chromosomal aneuploidy detector (UCAD) workflow. Primary liver cancer‐associated copy number changes (CNV) was identified from the discovery dataset (23 surgery‐ineligible patients and 41 health volunteers). A primary liver cancer (PLC) diagnosis method was then built based on CNV counts. This method was further prospectively validated on a cohort of 80 surgery‐eligible patients and 28 health volunteers.

### 3.2 Plasma cfDNA whole‐genome profiling identified 29 significantly altered chromosomal segments in liver cancer

**Figure 2:**
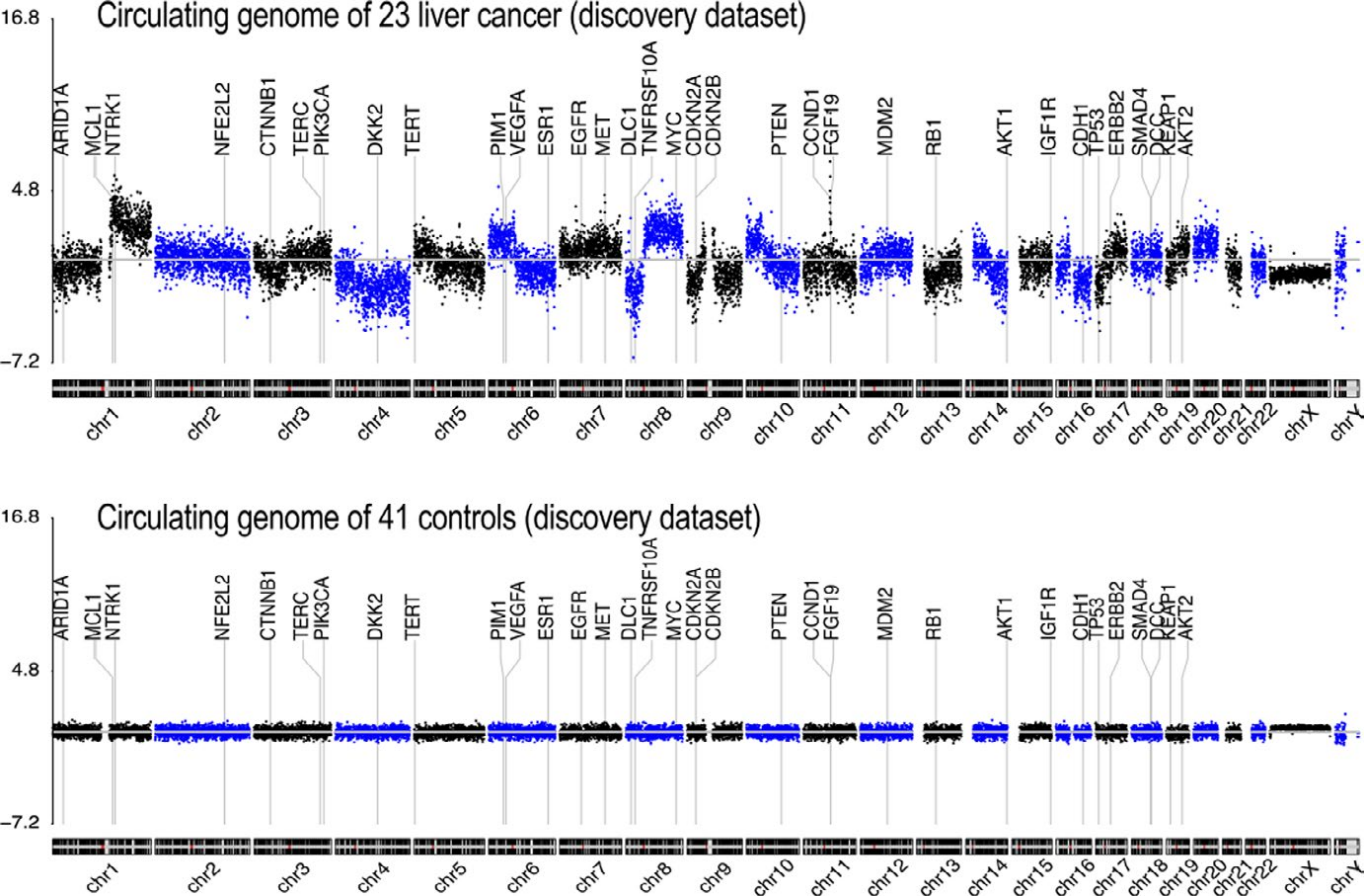
Circulating cancer genome of plasma cell‐free DNA from primary liver cancer patients. Circulating cell‐free chromosomal instability of 23 primary liver cancers (up) and 41 health controls (bottom). Chromosomes 1,2, …, and Y, are plotted from left to right. Each dot indicates the normalized coverage value of a 200K bin. Genes of interested are marked with dash lines.

Plasma samples were collected and profiled using the UCAD workflow before treatment. A genome‐wide overview of copy number variations is summarized in Figure 2. Chromosomal breakpoints were frequently identified on centromeres, resulting in chromosomal‐arm imbalances (Figure 2 top for averaged data plot). No significant CNV was found in healthy controls (Figure 2 bottom for averaged data plot). Chromosome arms, 1q, 6p, 7, 8q, 20, 10p, 5p15.33, 10cen, 15q, 17q, 19, 11q13.3, and 22q were identified as having statistically significant copy number gains in 78.26%, 47.83%, 34.78%, 91.30%, 52.17%, 30.43%, 39.13%, 21.74%, 17.39%, 39.13%, 8.70%, 13.04%, and 13.04% of samples, respectively, where well‐studied oncogenes, MYC (8q), MCL1 (1q), and VEGFA (6p), are located. Chromosome arms 1p, chr4, 6q, 10q, 13q, 8p, 11q, 11p, chr16, chr9, 17p, 21q, 14q, chr3, chr18, and 8pter were shown with statistically significantly copy loss in 65.22%, 60.87%, 47.83%, 60.87%, 65.22%, 52.17%, 39.13%, 43.48%, 43.48%, 47.83%, 52.17%, 30.43%, 43.48%, 21.74%, 26.09%, and 34.78% of samples, respectively, where potential tumor suppressor genes DLC1 (8p), DKK2 (4q), PTEN (10q), and TP53 (17p) are located (Table 1). Focal amplification on 5p15.33 (TERT) and 11q13.3 (CCND1, FGF19) was found in 7 (38.1%) and 3 (9.52%) liver cancer patients, respectively.

**Table 1:** Significant genomic changes in circulating liver cancer genome

Name	chrom	loc.start	loc.end	seg.mean	logP	Key genes	Frequency(%)
1p‐	chr01	0	145 000 000	−0.49	<=−100.0		65.22%
1q+	chr01	145 200 000	249 000 000	1.3568	<−100.0		78.26%
4‐	chr04	0	190 800 000	−0.7707	<−100.0		60.87%
6p+	chr06	0	69 000 000	0.7813	<−100.0	VEGFA	47.83%
6q‐	chr06	69 200 000	170 800 000	−0.5582	<−100.0		47.83%
7+	chr07	0	158 800 000	0.335	<−100.0		34.78%
8q+	chr08	43 600 000	146 000 000	1.0033	<−100.0		91.30%
10q‐	chr10	38 600 000	135 200 000	−0.4361	−69.3		60.87%
13q‐	chr13	19 000 000	114 800 000	−0.3688	−68.8		65.22%
8p‐	chr08	8 000 000	43 400 000	−0.9049	−62.1		52.17%
20+	chr20	0	62 400 000	0.4562	−57.1		52.17%
11q‐	chr11	71 600 000	134 800 000	−0.4313	−55.9		39.13%
11p‐	chr11	0	68 200 000	−0.4535	−46.3		43.48%
16‐	chr16	0	90 000 000	−0.4275	−45.5		43.48%
9‐	chr09	0	141 000 000	−0.2474	−38.1		47.83%
17p‐	chr17	0	19 800 000	−0.8408	−35.4	TP53	52.17%
21q‐	chr21	9 400 000	47 800 000	−0.4427	−29.1		30.43%
14q‐	chr14	19 000 000	107 000 000	−0.2948	−27.1		43.48%
10p+	chr10	200 000	35 600 000	0.318	−23.8		30.43%
3‐	chr03	0	197 800 000	−0.1392	−23.1		21.74%
18‐	chr18	0	77 800 000	−0.1713	−14.3		26.09%
TERT+	chr05	0	5 800 000	0.7377	−12.8	TERT	39.13%
8pter‐	chr08	0	6 800 000	−0.7036	−12.2		34.78%
10cen+	chr10	35 800 000	38 400 000	1.1605	−8.2		21.74%
15q+	chr15	20 000 000	102 200 000	0.119	−7.4		17.39%
17q+	chr17	20 000 000	80 800 000	0.1189	−6.3		39.13%
19+	chr19	0	58 800 000	0.123	−5.6		8.70%
CCND1+	chr11	68 400 000	71 400 000	1.1135	−5.5	CCND1	13.04%
22q+	chr22	16 000 000	51 000 000	0.0863	−3.2		13.04%

Note

Significant genomic changes were detected by binary circular segmentation. ‘chrom’, ‘loc.start’, and ‘loc.end’ define a chromosome segment which significantly changed. ‘seg.mean’ specified the normalized value of the segments. And ‘logP’ is the log‐transformed P value indicating how significant of the change by statistics. Results: Arm level changes were observed on chromosome 1, 4, 6, 7, 8, 20, 11, 16, 9, 17, 21, 14, 10, 3, 18, 15, 19. and 22. Focal amplification was observed on the region around CCND1 (chromosome 11q) and TERT (chromosome 5p).

Counts for altered segments in each patient are summarized in Table 2. The median number of mutated chromosomal segments for each sample was 11. Nineteen of 20 (95.7%) samples were found with at least 3 significantly altered chromosomal segments. One of 23 (4.3%) was found with no significantly mutated chromosomes. Meanwhile, 40 (97.7%) healthy controls exhibited no altered chromosomal segments at a Z‐score cut‐off of 2.702.

**Table 2:** Performance of UCAD as independent diagnosis

	Count of significantly changed chromosomal segments				
Discovery cohort (N = 64)					
#of segments	>=3	2	1	0	rate (>=1)
Healthy control (N = 41)	0	0	1	40	2.4%
Surgery ineligible liver cancer, before treatments (N = 23)	22	0	0	1	95.7%
Validation cohort: independent diagnosis (N = 108)					
#of segments	>=3	2	1	0	rate (>=1)
Presurgery plasma samples (N = 80)	32	10	13	25	68.8%
HCC (N = 63)	26	9	7	21	66.7%
ICC, ICC‐HCC mixed (N = 17)	6	1	6	4	76.5%
Health volunteer with follow‐up information (N = 28)	0	0	1	27	3.7%
Follow‐up cohort: disease and disease recurrence prediction					
#of segments	>=3	2	1	0	rate (>=1)
Postsurgery follow‐ups (19 pts, 29 samples)	2	1	7	19	31.0%

Note

Chromosomal arm level segment: z‐score>=2.702 and <=−2.702 as cut‐offs.

### 3.3 A method for primary liver cancer diagnosis based on cell‐free DNA CNV profiling

**Figure 3:**
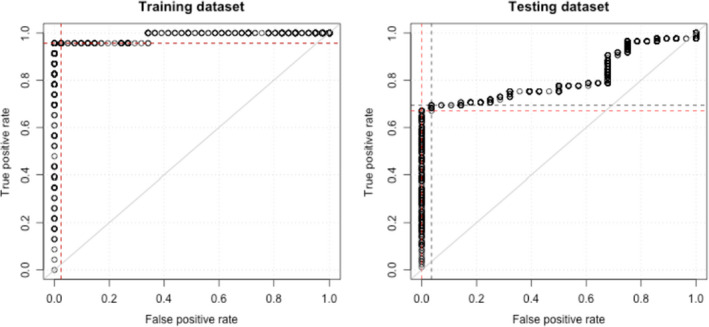
Performance of cfDNA CIN as independent diagnosis marker for PLC diagnoses. ROC curve plots with dash line indicates the false positive (cross at x‐axis) and true positive (cross at y‐axis) rate

Positive screening was defined by the cut‐off of ‘at least 1 significantly altered chromosomal segment’. A negative screening was defined as having no significantly altered chromosomal segment observed. The area under the curve (AUC) reached 0.99 and 0.85 in the training and validation datasets, respectively (Figure 3A,B).

An optimized cut‐off Z‐score = 2.702 was obtained by analyzing the ROC curve (Figure 3B). At the optimized cut‐off, the method reached 22/23 = 95.7% sensitivity and 40/41 = 97.7% specificity in the training dataset. While the method reached 55/80 = 68.8% sensitivity and 27/28 = 96.4% specificity in the validation dataset.

### 3.4 Independent diagnosis of 80 surgery‐eligible primary liver cancers

Copy number changes were further validated in a prospective cohort of 80 surgery‐eligible patients and 28 health volunteers. Plasma samples were collected before surgery and analyzed by UCAD, and each of the 29 segments were examined. Similar to the discovery dataset, frequent gains on chromosomes 7, 1q, and 8q were identified in the validation dataset with frequencies of 34.8%, 32.5%, and 31.2%, respectively. Frequent losses on chromosomes 6q, 17p‐, and 10q were identified with frequencies of 52.2%, 27.5%, and 25.0%, respectively. In summary, 55 of 80 (68.8%) samples were identified with positive screening with at least one significantly altered chromosome segments (Table 2). Among them, 42 (52.5%) samples were found with 2 or more significantly altered chromosomal segments. Thirteen (16.3%) samples were found with 1 significantly altered chromosomal segment. Further analyses identified positive screening in 42 of 63 (66.7%) hepatocellular carcinoma (HCC) cases, and in 13 of 17 (76.5%) intrahepatic cholangiocarcinoma (ICC) cases (Table 2). In contrast, 26 (96.3%) health volunteers were found with negative screening.

### 3.5 cfDNA CIN improves HCC diagnosis sensitivity in addition to AFP to detect surgery‐eligible PLC

**Figure 4:**
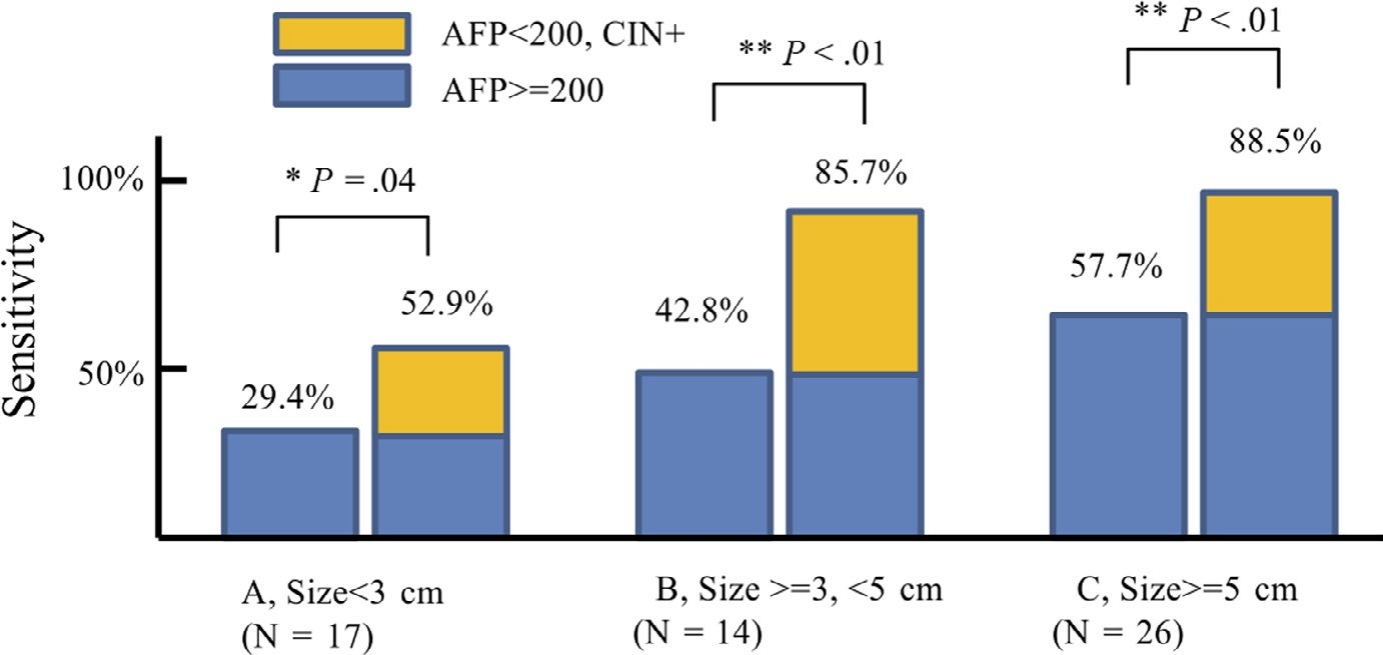
cfDNA CIN significantly improves HCC diagnosis performance in addition to AFP

In the validation dataset of surgery‐eligible patients, we compared cfDNA CNV results to AFP expression levels, which are used for hepatocellular carcinoma diagnosis. As shown in Figure 4, positive AFP (>=200) was found in 29.4%, 42.8%, and 57.7% hepatocellular carcinoma (HCC) patients with size less than 3 cm (Figure 4A), between 3 and 5 cm (Figure 4B), and greater than 5 cm (Figure 4C), respectively. Addition of CIN increases detection sensitivity to 52.9% (Figure 4A, tumor size less than 3 cm), 85.7% (Figure 4B, tumor size between 3 and 5 cm), and 88.5% (4C, tumor size greater than 5 cm), which is higher than for AFP alone (Fisher test, *P* = .04, <0.01, and < 0.01, respectively).

### 3.6 Microvascular invasion (mVI) is associated with plasma cfDNA CIN

Single‐parameter logistical regression analyses revealed that microvascular invasion (mVI) (OR > 10, 95% CI: [2.53,]), BCLC stage B/C (OR = 8.59, 95% CI: [1.07, 400]), and tumor size>=3 cm (OR = 5.68, 95% CI: [1.45, 28.1]) are associated with detectable copy number changes (Table 3B). All other parameters, including histology, age, and AFP level, were not statistically significant (Table 3A).

**Table 3:** Clinicopathological parameters mVI correlates with UCAD positivity

A,							
	Number of statistically changed chromosomes (N = 80) (Z‐score cutoff = 2.702)					Fisher exact test	
	>=3	2	1	0	% (>=1)	OR (>=1 vs other)	*P*
Age							
>=56y	11	5	8	13	64.9%		NS
<56y	19	2	5	10	72.2%		
NA	2	3	0	2	71.4%		
Gender							
Male	26	6	11	20	67.2%		NS
Female	4	1	2	3	70.0%		
NA	2	3	0	2	71.4%		
Histology							
HCC	26	9	7	21	66.7%		NS
ICC, HCC‐ICC	6	1	6	4	76.5%		
Size							
>=5 cm	23	3	6	6	84.2%	5.50 [1.70‐20.3]	0.002
>=3 cm, <5 cm	6	1	2	8	52.9%		
<3 cm	1	3	4	10	44.4%		
NA	2	3	1	1	85.7%		
Count of tumor							
Single	19	6	9	21	61.8%		NS
Multiple	9	1	1	3	78.6%		NS
NA	4	3	3	1	90.9%		
Tumor encapsulation							
Complete	17	10	7	17	66.7%		NS
Incomplete	13	0	3	10	61.5%		NS
No	1	0	0	1	50.0%		NS
NA	1	3	0	2	66.6%		
Satellite nodules							
0	22	6	8	15	70.6%		NS
1	3	2	3	7	53.3%		NS
>1	5	2	1	1	88.9%		NS
NA	2	0	1	2	60.0%		
HBV DNA							
<=10 000	5	2	2	5	64.3%		NS
>10 000	19	5	10	11	75.6%		NS
NA	8	3	1	9	57.1%		
Alanine aminotransferase							
<=41	22	4	9	15	70.0%		
>41	9	3	4	9	64.0%		NS
NA	1	3	0	1	80.0%		

B,							
	Number of statistically changed chromosomes (N = 63) (Z‐score cutoff = 2.702)					Fisher exact test	
	>=3	2	1	0	% (>=1)	OR (>=1 vs other)	*P*
mVI							
M0	8	3	5	11	59.3%	‐	
M1	3	2	1	9	40.0%		
M2	12	1	1	0	100%	>10 [2.53‐Inf]	8.7e‐4
NA	3	3	0	1	85.7%		
Size (cm)							
<3	1	2	4	10	41.2%		
>=3, <5	5	1	2	6	57.1%		
>=5	18	3	1	4	84.6%	5.68 [1.45‐28.1]	5.6e‐3
NA	2	3	0	1	83.3%		
AFP (ug/L)							
<20	10	2	1	9	59.1%		
20 200	1	0	4	4	55.6%		
>=200	13	4	2	7	73.1%		NS
BCLC Stage							
B,C	10	2	0	1	92.3%	8.59 [1.07‐400]	4.0e‐2
A	14	3	6	17	57.5%		
NA	2	4	1	2	‐		

All parameters were also analyzed by multiple‐parameter logistical regression. As shown in Table 4, mVI was the only statistically significant parameter. Neither BCLC stage nor tumor size reached statistical significance. Results indicate that mVI might be the major factor associated with detectable copy number change in plasma cfDNA.

**Table 4:** MVI is the independent predictor of UCAD positivity

	*P* value	Odds ratio	95% CI
mVI = M2	.044	10.7	1.31, 152.9
Tumor size > 3cm	.142	5.53	0.771, 113
BCLC stage B or C	.755	1.43	0.134, 15.3
AFP>= 200	.274	0.350	0.0404, 1.95
Age>= 56	.201	0.589	0.125, 2.77

### 3.7 UCAD‐positive screening is associated with PLC recurrences

**Figure 5:**
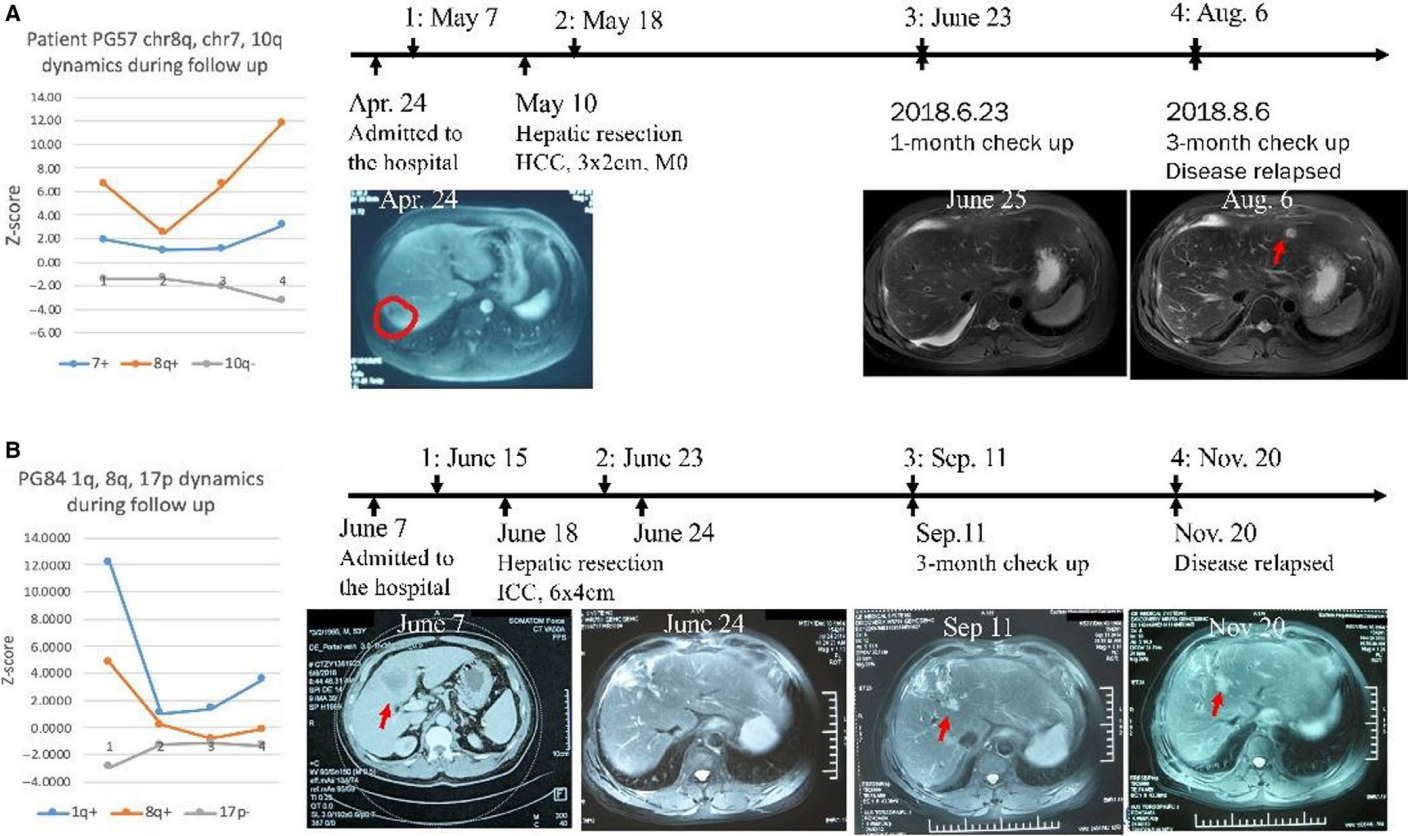
Plasma cfDNA CIN is associated with disease recurrence for patients after R0 resection. A, Patient PG57 (HCC confirmed by pathological examinations) shows continuing changes of chromosome 8q, 7 and 10q after surgery (A, left). Imaging diagnoses for (or close) to each time point were presented (A, right). B, Patient PG84 (ICC confirmed by pathological examinations) shows continuing changes of chromosome 8q, 7 and 17p after surgery (B, left). ). Imaging diagnoses for (or close) to each time point were presented (B, right)

Twenty‐nine plasma samples were collected for follow‐up from 19 postsurgery liver tumors. Nine (31.0%) postsurgery samples from 6 (31.5%) patients were found with positive screening during follow‐up. Three (50.0%) positive screening patients were then confirmed as having disease recurrences within the 6‐month follow‐up period. For patient PG57, blood testing showed significant 8q gain (Z‐score = 6.63) before surgery (Figure 5A). Pathological examination confirmed a 3x2 cm hepatocellular carcinoma with no microvascular invasion after R0 tumor resection. A plasma sample was collected again 10 days after surgery. The 8q Z‐score was 2.46, which was still statistically significant, indicating potential tumor residuals and risk of relapse. The patient was assessed again 1 month later, and a plasma sample was collected. UCAD testing showed that 8q Z‐score was back to 6.58, indicating potential tumor relapses. Subsequent MRI/CT imaging confirmed tumor relapsed. The 8q Z‐score continued increasing to 11.8 in the last visit, indicating tumor progression. Similarly, for patient PG84, the 1q Z‐score was 12.1 before surgery. The Z‐score went down to 1.05 after surgery, however, the 1q Z‐score continued to increase back to 3.50 during follow‐up (Figure 5B). PG84 was subsequently confirmed as having tumor relapse.

## 4 DISCUSSIONS

Primary liver cancer (PLC) is responsible for a high incidence of cancer‐related mortality worldwide. However, even in good surgical candidates, long‐term survival rates remain unsatisfactory due to high recurrence rates. In this study, significant chromosomal instability (CIN) and copy number variations (CNV) were found in plasma cell‐free DNA of 69.8% resectable liver cancers (Table 2). This research also shows that plasma cfDNA copy number variation is independent of AFP expression level (Table 3B, Figure 4A,B,C). Positive serum AFP at cut‐off 200 µ g/µL was found in 47.4% of patients, consistent with previous reports.^21^ UCAD alone exhibited higher positive rates (69.8%, *P* < .05), and addition of UCAD to AFP(>200 µ g/µL) identified 84.2% surgical‐eligible hepatocellular carcinomas, including 52.9%, 85.7%, and 88.5% hepatocellular carcinomas with size less than 3 cm (Figure 4A), size between 3 and 5 cm, and tumor larger than 5 cm, respectively, which is significantly higher than that of AFP alone at cut‐off 200 ng/mL (29.4%, 42.8%, and 57.7%, respectively). These results suggest simultaneous use of UCAD testing and plasma serum protein level biomarkers may identify more cases of hepatocellular carcinoma.^4^, ^22^ The performance of UCAD and CA199/CEA to identify non‐HCC liver cancers still needs further investigations.

The presence of microvascular invasion (MVI) has been reported as one of the most important risk factors related to postsurgery tumor recurrence. In a previous report,^17^ anatomic resection had significantly better overall and disease‐free survival rates than limited resection in solitary small HCC (≤5 cm) with MVI. Liver transplantation can potentially improve patient survival for patients with low MVI compared to hepatic resection.^18^ In this study, we find that positive plasma cfDNA copy number variation screening is associated with MVI. All M2 (high risk for MVI) patients were found with UCAD‐positive screening, while all UCAD‐negative patients were either M0 or M1 (low risk for MVI). These results suggest that UCAD screening may serve as a surrogate biomarker of MVI, for optimizing treatment options.

Furthermore, positive screening was found in 6 patients after R0 resection, 3 of whom were confirmed as having disease relapse during follow‐up. These results suggest that plasma cfDNA copy number variations might help identify patients who are at risk for residual disease and disease relapses. Postsurgery adjuvant therapies, including TACE, may be suggested for those patients, since it was shown to improve patient survival in previous studies.^23^ Further clinical studies are still needed to confirm the survival benefits for these patients. Tumor heterogeneity and clonal evolution are potential major causes of resistance to treatment. In this research, patient PG57 exhibited a continuing gain of chromosome 7q, especially on the region of the oncogene c‐Met, which may indicate a selection pressure on cMet‐amplified clones. A cMet inhibitor, such as Crizotinib,^24^ along with conventional therapies may help improve patient survival.

Chromosomes changes may also be detectable in HBV carriers. In previous reports,^5^ chromosome 1q and 8q gains were detected in 3/67 (4.5%) and 8/36 (22.2%) HBV carrier patients without and with cirrhosis, respectively. HCC was diagnosed several months after the blood collection for some of the patients. It is likely that the cancer would have been present at the time of blood collection and was associated with the CNAs in plasma. Epidemiology studies also showed that the 5‐year cumulative HCC risk is about 1.5%‐3% and 15% for HBV carriers without and with cirrhosis, respectively.^25^ Hence, a prospective study involving HBV carriers is need to further validate the utility of cell‐free DNA in early hepatocellular carcinoma screening, especially the sensitivity and specificity of HCC detection ahead of imaging diagnoses.

In summary, the preliminary data in this study suggest that plasma cell‐free DNA chromosomal instability analysis may aid HCC surveillance, assessments of treatment response, and prognosis.

All data generated or analyzed during this study are included in this article.

## CONFLICTS OF INTEREST

The authors disclose no conflicts.

## AUTHOR CONTRIBUTIONS

Study concept and design: Minggen Hu, Yan Meng, and Nan Li. Sample collection and Acquisition of data: Cunzhen Zhang, Haibei Xin, Guanghui Ding, Jin Wang, Zhiwen Ding, Shanshan Li, and Shuang Feng. Analysis and interpretation of data: Shuang Feng, Zhiwen Ding, Jin Wang, Ziliang Qian, and Shupeng Liu. Drafting of the manuscript: Shuang Feng, Zhiwen Ding, and Jin Wang. Statistical analysis: Shuang Feng, Zhiwen Ding, and Jin Wang. Study supervision: Minggen Hu, Yan Meng, and Nan Li.

## Supporting information

Supplementary Material

## Data Availability

The data used to support the findings of this study are available from the corresponding author upon request.
