# Supplementary material for: Investigation of Plasma cell‐free cancer genome chromosomal instability as a tool for targeted minimally invasive biomarkers for primary liver cancer diagnoses
Source: Cancer Med. 2020 May 27;9(14):5075–85. doi: 10.1002/cam4.3142 (PMC7367647; doi:10.1002/cam4.3142)
Supplement: Supplementary file 1 — Supplementary Material [file CAM4-9-5075-s001.docx]

Supplementary Table 1. Baseline information for 3 cohorts

|  | Discovery cohort | | Validation cohort | | Disease prediction cohort | |
| --- | --- | --- | --- | --- | --- | --- |
|  | Cancer patients | Healthy volunteers | Cancer patients | Healthy volunteers | Cancer patients | Cirrhosis patients |
| Median age | 55 | 56 | 55 | 48 | 52 | ? |
| Gender(Male%) | 77% | 60% | 89% | 51% | 69% | 100% |
